# Tumor and Stromal-Based Contributions to Head and Neck Squamous Cell Carcinoma Invasion

**DOI:** 10.3390/cancers7010382

**Published:** 2015-02-27

**Authors:** Steven M. Markwell, Scott A. Weed

**Affiliations:** Department of Neurobiology and Anatomy, Program in Cancer Cell Biology, Mary Babb Randolph Cancer Center, West Virginia University, Morgantown, WV 26506, USA; E-Mail: smarkwell@hsc.wvu.edu

**Keywords:** invasion, HNSCC, tumor microenvironment, metastasis

## Abstract

Head and neck squamous cell carcinoma (HNSCC) is typically diagnosed at advanced stages with evident loco-regional and/or distal metastases. The prevalence of metastatic lesions directly correlates with poor patient outcome, resulting in high patient mortality rates following metastatic development. The progression to metastatic disease requires changes not only in the carcinoma cells, but also in the surrounding stromal cells and tumor microenvironment. Within the microenvironment, acellular contributions from the surrounding extracellular matrix, along with contributions from various infiltrating immune cells, tumor associated fibroblasts, and endothelial cells facilitate the spread of tumor cells from the primary site to the rest of the body. Thus far, most attempts to limit metastatic spread through therapeutic intervention have failed to show patient benefit in clinic trails. The goal of this review is highlight the complexity of invasion-promoting interactions in the HNSCC tumor microenvironment, focusing on contributions from tumor and stromal cells in order to assist future therapeutic development and patient treatment.

## 1. Introduction

Head and neck squamous cell carcinoma (HNSCC) is one of the most aggressive and invasive cancer types [1]. A common HNSCC hallmark is loco-regional invasion and metastasis to cervical lymph nodes, accounting for an 88% patient mortality rate in the two years following metastatic disease development [1]. Despite the long-held notion of genomic instability in advanced disease stages, recent studies have found no difference in the accumulation of mutations in tumors from patients with and without lymph node involvement [2]. This indicates that alterations other than mutations in signaling pathways likely account for progression from primary tumor to invasive and metastatic disease. The contributions towards metastatic disease arise both from changes in the behavior of tumor cells and interactions with various stromal components in the tumor microenvironment. The purpose of this review is to highlight the molecular and cellular mechanisms utilized by tumor cells and the associated microenvironment in promoting HNSCC invasiveness.

## 2. Tumor Cell Contributions

In HNSCC patients, two out of three individuals exhibit locoregional or distal metastasis at diagnosis, correlating with poor patient survival [3,4]. As in other carcinomas, HNSCC invasion involves a multi-step process that entails initial breaching of the basement membrane, tumor cell migration through the stromal extracellular matrix (ECM), intravasation into regional vasculature, and extravasation at the metastatic site. These stages frequently utilize proteolytic-mediated degradation of ECM proteins to facilitate tumor cell spreading [5,6,7,8,9].

### 2.1. Cell-ECM Interactions

The activity of several actin cytoskeletal-modulating proteins has been demonstrated to alter the invasive nature of HNSCC. The basement membrane and ECM are barriers that tumor cells must bypass in order to move into the surrounding stroma [6,10,11]. Tumor cell mediated proteolytic degradation of ECM components, globally or at focalized points termed invadopodia, is essential to the invasive process [6,10,11,12]. Invadopodia are actin-based membrane protrusions that mediate tumor cell dissemination by degrading restrictive ECM proteins through the action of matrix metalloproteinases (MMPs) [5,10,11,12]. Many MMPs are overexpressed in HNSCC, including the invadopodia-associated MMPs MMP-2, MMP-9, and MMP-14 [9,12,13]. Invadopodia comprise a central filamentous (F)-actin core surrounded by an integrin-based adhesion complex ring [6,10,11]. Cortactin and Arp2/3 complex are essential protein components involved in formation of the F-actin invadopodia core [14,15,16,17]. Cortactin is overexpressed in several cancer types including HNSCC, resulting in enhanced tumor cell motility and invasion [18,19,20,21,22,23,24,25]. Cortactin stabilizes actin branch points, binding to both the F-actin “mother” filament and Arp2/3 complex on the “daughter” filament [22,25,26,27]. The end result of this activity is enhanced invadopodia formation and maturation, leading to robust localized ECM degradation [22,25,26,27]. Further evidence indicates that cortactin overexpression correlates with lymph node involvement and metastases [28,29,30]. In addition to modulating cytoskeletal dynamics, cortactin facilitates localization and activation of MMP-14 (also termed membrane type 1—matrix metalloproteinase (MT1-MMP)) to invadopodia along with the secretion of MMP-2 and MMP-9 at sites of focalized degradation of ECM proteins [13,31,32]. The activity of MMP-14, MMP-2, and MMP-9 is significantly elevated in HNSCC cell lines with high metastatic potential and well as oral cancer patient samples with lymph node involvement [12,33,34].

Several studies have demonstrated increased localization of the actin bundling protein fascin at the tumor invasive front [35,36]. Facin functions by bundling F-actin, which facilitates the formation of cellular protrusions necessary for cell-ECM interactions and cell motility [35,36,37]. Bundling of F-actin into parallel strands stabilizes filopodia and invadopodia, resulting in enhanced cell motility and localized ECM degradation [35,36,37]. Re-expression of fascin in facin-null SW1222 human colonic epithelial cells results in relocalization of integrin β1 and vinculin to the leading edge of motile cells [38]. Overexpression of fascin in various tumors, including HNSCC, correlates with aggressive disease, high metastatic potential, and poor prognosis [35,36].

Similarly, the serine/threonine kinase p21 protein (Cdc42/Rac)-activated kinase (PAK1) is enriched at the invasive boarder of HNSCC tumors, and is essential for HNSCC invasion *in vitro* [39,40]. PAK1 resides in the cytoplasm, but can be detected at the leading edge of motile cells, focal adhesions, cell-cell junctions, and cortical actin structures [41,42,43,44]. PAKs phosphorylate several cytoskeletal protein targets, including vimentin, desmin, LIM kinase (LIMK), myosin light chain (MLC), and myosin light chain kinase (MLCK), where phosphorylation directly correlates with enhanced cellular motility [39,40]. PAK1-mediated MLCK phosphorylation reduces stress fiber formation, while PAK-1-mediated MLC phosphorylation induces contractility [41,45,46]. LIMK activation facilitates LIMK binding to the F-actin severing protein ADF/cofilin, inhibiting ADF/cofilin activity via phosphorylation to stabilize the F-actin network [41,47,48]. The p41-ARC subunit of Arp2/3 complex can be directly phosphorylated by PAK1, activating Arp2/3 actin nucleation activity to enhance F-actin formation and increase cell motility [49,50]. This effect on actin network formation can also be accomplished through PAK1 phosphorylation of cortactin [49,51]. In addition to altering cytoskeletal dynamics, PAK1 has been implicated in the downregulation of cell-cell contacts. PAK1-mediated phosphorylation of the transcription factor Snail results in reduced expression of the epithelial cell-cell adhesion molecule epithelial (E)-cadherin [41,52]. Secretion of MMP-1, MMP-3, and MMP-9 correlates directly with PAK1 expression, suggesting that the activity of PAK1 may enhance proteolytic degradation of ECM [53,54]. Overexpression of PAK1 in various tumors, including HNSCC, correlates with aggressive disease and poor prognosis [39,40].

The calcium binding proteins S100A8 and S100A9 belong to a family of low-molecular-weight cytoplasmic proteins primarily detected as a S100A8/A9 heterodimer termed calprotectin [55,56,57,58]. Expression and secretion of S100A8/A9 is associated with chronic inflammation and is released from tumor cells in response to hypoxic stress [55]. While S100A8 and S100A9 are overexpressed in a multitude of cancers, their expression is suppressed in HNSCC [55,59,60]. Certain studies have demonstrated a pro-apoptotic role of S100A8/A9, inducing pro-caspase-3 cleavage and downregulating expression of anti-apoptotic members of the Bcl family, Bcl2 and Bcl-X_L_ [55,61]. The ability of S100A8/A9 to induce an apoptotic response, rather than the role in inflammatory signaling, is the most likely reason that expression of these proteins is lost in HNSCC. In addition to inflammatory signaling and apoptotic response, S100A8/A9 regulates the expression and secretion of MMP-2, representing a potential upstream therapeutic target [59,60]. Thus, calprotectin may serve a dual role in HNSCC by preventing apoptosis while facilitating MMP-2-driven metastatic dissemination.

In order to monitor the surrounding ECM, cells form actin-rich protrusions that in a migratory cell contact the ECM to form structures known as focal adhesions. Focal adhesions contain the well-characterized cytoskeletal proteins talin, paxillin, α-actinin, vinculin and focal adhesion kinase (FAK) [62,63,64]. Focal adhesions serve as intermediary structures by linking the actin cytoskeleton within the cell to the ECM surrounding the cell by interacting with the cytoplasmic domains of the integrin class of transmembrane ECM receptors [62,65,66,67,68]. Integrin extracellular domains directly bind ECM proteins, including fibronectin, laminin, collagen I and collagen IV. [62,65,66,67,68]. FAK activation precedes focal contact formation and facilitates focal adhesion maturation through phosphorylation of Rho guanine nucleotide exchange factors and phosphatidylinositol phosphate kinase isoform γ, which enhances talin binding to integrin cytoplasmic domains [66,69]. Regulation of focal adhesion disassembly at the trailing edge by FAK dramatically alters cellular motility [66,70,71]. FAK overexpression occurs early in HNSCC development, correlating with increased tumor cell invasion and lymph node metastasis, partially through an increase in MMP-2 and MMP-9 secretion [67,68,69]. As such, FAK has become a therapeutic target in many tumor types, where pharmacological inhibition of FAK tyrosine kinase activity results in decreased tumor cell invasion [72,73,74,75].

Phospholipase D (PLD1), mediates the hydrolysis of phosphatidyl choline into choline and the second messenger phosphatidic acid [49,76,77]. Phosphatidic acid is further hydrolyzed by phosphatidic acid phosphohydrolases to generate diacylglycerol and lysophosphatidic acid (LPA), the latter being a key mediator of inflammatory response and has been implicated in oncogenesis and metastatic progression [10,76]. In addition, LPA activates the Rho family of cytoskeletal regulatory GTPases, facilitating the formation of filopodia, lamellipodia, and stress fibers essential for cell movement [49,76]. PLD1 has been shown to drive stress fiber and focal adhesion formation in HeLa cells [78]. PLD1 is overexpressed in several cancers including HNSCC, where it activates Src kinase and mitogen activated protein kinase (MAPK), driving invadopodia formation, maturation, and tumor cell invasion [79,80,81,82]. Due to the numerous migratory and invasive signaling networks stimulated by PLD1 and PLD1 substrates, PLD1 represents a viable upstream target for limiting tumor spread and metastatic progression. To this end, the PLD1 inhibitors quercetin, ML298, and ML299 decrease U87 glioblastoma cell invasion in *in vitro* assays [83,84]. These data support further investigation into PLD1 inhibitor efficacy in suppressing HNSCC invasion.

The phosphoinsositide-3-kinase (PI3K) family of kinases are among the most frequently altered oncongenic drivers in cancer [85,86]. Genomic alteration of PI3K occurs in approximately 31% of HNSCC tumors [85,86]. The PI3K class IA isoforms, p110α, p110β, and p110δ lie directly downstream of many oncogenic receptor tyrosine kinases, including epidermal growth factor receptor (EGFR), human epidermal growth factor receptor 3 (HER3), Met, platelet-derived growth factor receptor (PDGFR), vascular endothelial growth factor receptor (VEGFR), and insulin-like growth factor receptor 1 (IGF-1R) [85,87]. The PI3K isoform p110α is the most commonly overexpressed family member in HNSCC, acting upstream of Cdc42, Rac, and Rho kinases, to enhance filopodia and lamellipodia formation resulting in increased cellular motility [85,86,88,89,90].

Despite the expression of several fibroblast growth factor (FGF) receptors in HNSCC, surprisingly little investigation has focused on secretion of the FGF gene products FGF-3, FGF-4, and FGF-19 located within the 11q13 amplified region found in nearly a third of HNSCC patient samples [91,92]. Studies have focused on FGF-2 and FGF-binding protein, identifying autocrine loops with these FGF receptors that correlate with enhanced HNSCC invasion [92,93]. Given the establishment of these autocrine loops and the potential for these secreted FGFs to attract fibroblasts into proximity with HNSCC cells (see below), further investigation into the 11q13 amplified FGFs is warranted to determine if these proteins contribute to HNSCC metastatic progression.

### 2.2. Cell-Cell Interactions

In addition to enhanced motility at the individual cellular level, the mode of tumor cell migration also impacts local invasion and metastasis. Tumor cells can invade as individual cells, displaying either mesenchymal or amoeboid migration depending on intercellular signaling events, which result in poorly differentiated tumors due to the intermingling of individual invasive tumor cells with the stromal tissue [94,95]. Other tumor cells utilize multicellular or collective invasion, maintaining tumor cell-cell junctions, resulting in moderately to well differentiated tumors as the invasive tumor cells can be distinguished from the surrounding tissue [94,95]. In histological HNSCC samples displaying a broad invasive front, tumors remain well-to moderately-differentiated due the tumor cells being easily distinguished from the surrounding tissue by retaining membranous E-cadherin staining. These characteristics indicate that such tumors undergo collective invasion. In addition, cases where tumors display individual finger-like invasive fronts, tumors are poorly differentiated as individual tumor cells are intermingled with stromal cells. These invasive tumor cells show reduced E-cadherin staining, with notable increases in both phospho-Src and vimentin that represent a more mesenchymal invasion modality [96]. Patients with elevated phospho-Src and vimentin have direct correlation with greater lymph node involvement and advanced tumor stage [96]. Although E-cadherin is not essential to collective invasion, maintenance of cell-cell adhesions and an epithelial phenotype allow for multicellular invasive clusters to migrate simultaneously [94,95].

In addition to direct cell-cell contact, tumor cells interact through autocrine and paracrine signaling networks. EGFR is overexpressed in greater than 95% of HNSCC patient samples, and phosphorylation of the downstream effector Src kinase correlates with poorly differentiated HNSCC, lymph node involvement, and poor patient outcome [97,98,99]. Recent studies indicate that there are two distinct subpopulations within most HNSCC tumors, in which E-cadherin and vimentin are inversely expressed [100,101,102]. These two subpopulations demonstrate plasticity in regenerating heterogeneity in culture and xenograft tumors derived from single subpopulations, but respond differentially to various chemotherapeutic agents [100,101,102]. Expression of EGFR is variable in these subpopulations, correlating inversely with vimentin expression, suggesting a potential mechanism for acquired EGFR inhibitor resistance that is observed in the clinic [100,101,102]. Another receptor tyrosine kinase, tyrosine receptor kinase B (TrkB), is expressed in more than half of HNSCC patient tumors. TrkB activates the transcription factors Snail and Twist, driving the epithelial to mesenchymal transition (EMT) and enhancing tumor cell invasion [103]. These data collectively support the idea that deterioration of cell-cell contacts drives a drug resistant and more invasive phenotype in HNSCC.

### 2.3. Angiogenesis and Neo-Vascularization

Angiogenesis not only supplies growing tumors with requisite nutrition, but also provides cells at the tumor periphery a route to disseminate into surrounding tissues and the rest of the body. In addition to MMPs, HNSCC cells secrete a variety of pro-angiogenic factors that recruit endothelial cells into the local tumor microenvironment, resulting in formation of a leaky capillary bed that facilitates tumor cell intra- and extravasation. Two key angiogenic paracrine signaling profiles have been proposed for HNSCC cells. The first utilizes excess secreted VEGF, FGF-2, with small amounts of interleukin (IL)-8. The second mainly consists of IL-8, with lesser amounts of VEGF and FGF-2 [104,105,106]. In addition, primary HNSCC tumor cell cultures, tissue specimens, and established cells lines have enhanced secretion of VEGF and/or PDGF-AB, with lesser, yet still elevated, secretion of granulocyte colony stimulating factor (G-CSF) and granulocyte macrophage (GM)-CSF [105,107]. Increased secretion of these cytokines drives HNSCC tumor angiogenesis and corresponds with decreased patient survival [105]. Furthermore, oral SCC tissue samples display enhanced lymphatic microvessel density in the presence of VEGF, PDGF, basic FGF, hepatocyte growth factor (HGF) and IGF-1 [108,109]. Enhanced primary tumor lymphatic and blood microvessel density in response to these secreted factors correlates with lymph node metastasis and invasive tumor margins [110,111]. Endothelial cell recruitment and formation of an immature vascular network around the tumor in response to HNSCC cell angiogenic secretions are therefore prime contributors for providing essential routes for primary tumor cell invasion and metastatic dissemination.

### 2.4. Metastasis to Distant Sites

Once tumor cells reach the blood or lymphatic vasculature, they must survive in circulation until they reach lymph nodes or other metastatic sites. While little has been elucidated about such circulating tumor cells (CTCs) in HNSCC, the amount of HNSCC CTCs rises significantly in stage IV tumors, correlating directly with increased metastasis and inversely with therapeutic response [112,113]. HNSCC CTCs are not well defined, and are typically characterized as cells expressing epidermal cell adhesion molecule (EpCAM) or cytokeratin (CK) 8, CK18, or CK19 in blood samples [112,113]. One study found that IL-6 enhanced survival and self-renewal of the aldehyde dehydrogenase (ALDH)^high^CD44^high^ cell population, representing a potential cancer stem cell (CSC) subpopulation sufficient to reconstitute a tumor when transplanted into a mouse xenograft model [114]. This same CSC subpopulation is resistant to cisplatin-induced cell death [115]. There is evidence that indicates EGFR, TrkB, and IL-1β are essential to maintaining a mesenchymal subpopulation associated with chemotherapeutic resistance in HNSCC [103,116,117]. Other studies suggest that these mesenchymal-like cells can recapitulate the epithelial population of a tumor following chemotherapeutic therapy, potentially representing the HNSCC tumor equivalent to the CD44^+^/CD24^−^ stem-like subpopulation in breast carcinomas [100,101,102]. It remains unclear if these mesenchymal-like cells, CSCs and CTCs are the same or unique HNSCC subpopulations, but all show tumor initiating capacity that can be utilized to form metastases [101,102,103,114,115,117]. Once these tumor initiating cells (TICs) reach the metastatic site, they must first extravasate, a process aided by the local endothelial cells [114]. Following extravasation, some TICs differentiate back into the more epithelial phenotype that makes up the majority of the tumor mass, while other TICs undergo self-renewal to maintain the subpopulation [100,101,102,114]. Reconstituting the entire tumor mass allows the tumor to grow rapidly, taking advantage of the hospitable metastatic niche since the epithelial cell phenotype shows enhanced proliferation rates as compared to TICs [101,117]. While CTCs, CSCs, and mesenchymal-like cells represent resistant subpopulations potentially capable of initiating recurrence and correlates with invasive and metastatic disease, investigation into this aspect of HNSCC progression for therapeutic targeting has become an important newly emerging field [100,103,112,113,116].

## 3. Stromal Cell Contributions

In addition to carcinoma cells, various cellular and acellular stromal components contribute to promoting and maintaining HNSCC invasion. Deposition of specific ECM proteins (collagen IV, collagen XVII, fibronectin, and laminin) is enhanced in HNSCC tumors and serve as a chemo-attractant for HNSCC cells in various *in vitro* invasion assays [118,119,120]. As HNSCC tumors progress towards metastatic disease, non-tumor cell types from the associated stroma have been shown to have direct and indirect roles in facilitating HNSCC invasion.

### 3.1. Mast Cells

Mast cells are part of the immune myeloid lineage that mediate innate and acquired immune responses through granule exocyctosis, releasing histamine, serine proteases, carboxypeptidase A (CPA1), proteoglycans, prostaglandin D_2_ (PGD_2_), leukotriene C_4_ (LTC_4_), tumor necrosis factor (TNF)-α, GM-CSF, IL-3, IL-4, IL-5, IL-6, IL-8, and IL-16 [121]. During advanced HNSCC stages, where the tumor has spread to loco-regional or distal lymph nodes, mast cells accumulate in the tumor stroma, with their presence directly correlating with increased angiogenesis [104,122,123]. How HNSCC tumors suppress rapid mast cell activation in response to immunoglobulin E or CD32 binding to F_cε_RI or F_Cγ_RIIb respectively remains to be elucidated, but may occur by blocking F_cε_RI activation on mast cells [121]. Additionally, heparanase, an enzyme involved in cleavage and remodeling heparin sulfate proteoglycans in the ECM, accumulates at the HNSCC invasive front, and is a marker of poor prognosis for lymph node metastasis and tumor recurrence [124]. Mast cells, along with tumor infiltrating neutrophils, endothelial cells, and macrophages exhibit heparanase activity [124,125]. However, since mast cells also secrete large amounts of heparin, they are the cell type that is likely responsible for invasion-associated heparanase activity in the tumor microenvironment. While the main contribution of mast cells to tumor progression may be inflammation-mediated recruitment of other cell types into the microenvironment, their presence also facilitates HNSCC tumor neo-vascularization and dissemination to loco-regional lymph nodes.

### 3.2. Neutrophils

Neutrophils are another component of the immune system that contributes to the innate immune response [126]. Neutrophils are recruited to the tumor microenvironment by pro-inflammatory signals, including IL-8, transforming growth factor (TGF)-β, IL-4, IL-10, IL-13, GM-CSF, and TNF-α [127,128]. Following recruitment to the tumor microenvironment, neutrophils secrete VEGF-A, stimulating neovascularization through endothelial cell recruitment and proliferation, which can be abrogated via anti-VEGF-A antibodies or angiostatin treatment [128,129,130]. Additionally, neutrophil-derived HGF and MMP-9 facilitate tumor cell migration and invasion towards the newly formed vascular bed [128]. In this context, neutrophils bridge the gap between the growing tumor mass and the local vasculature, bringing in endothelial cells to an area of growing hypoxia while promoting a chemotactic invasive phenotype in the tumor cells.

### 3.3. Macrophages

Macrophages belong to the myeloid lineage of the immune system [131]. Macrophages play a direct role in immune surveillance through endocytosis of pathogens and cellular debris [131]. Tumor associated macrophage (TAM) infiltration into the tumor microenvironment correlates with lymph node involvement, tumor stage, and extracapsular spread [132,133,134]. Once TAMs arrive in the tumor microenvironment, TAM secretions set up several paracrine signaling loops that drive tumor cell invasion and metastasis. In one loop, TAMs secrete EGF stimulating tumor cell growth, migration, and invasion. Correspondingly, HNSCC cells secrete CSF-1 that drives further TAM proliferation and tumor infiltration [135,136,137]. In another loop, TAMs secrete macrophage migration inhibitory factor (MMIF), attracting and activating neutrophils, which subsequently interact with HNSCC cells as described above [128,129,130,138]. In response to HNSCC secreted paracrine factors, TAMs develop podosomes, capable of assisting tumor cells breach the basement membrane and enter the vascular or lymphatic network [133,139]. Similar to invadopodia, podosomes are membrane protrusions containing an actin-rich core surrounded by an integrin ring that mediates interaction with the ECM [140]. Podosomes are formed at the leading edge of motile cells and contribute to cellular motility, simultaneously allowing cells to adhere to the ECM and initiating acto-mysoin contractility to pull the cell body forward [139,140]. Podosomes can also localize MMPs, including MMP-2, MMP-9, and MT1-MMP to proteolytically degrade and rearrange the ECM [140,141]. TAMs also secrete the chemotactic factor macrophage inflammatory protein (MIP)-3α, which drives HNSCC cell migration and invasion [142]. Through these signaling pathways, macrophages are able to promote and maintain the HNSCC invasive phenotype, assist in basement membrane breakdown and recruitment of other cell types into the tumor microenvironment.

### 3.4. Endothelial Cells

While endothelial cells play a major role in vascularization of the growing tumor mass, emerging evidence demonstrates a novel role for endothelial cells in facilitating tumor cell invasion. The chemotactic factors VEGF, TNF-α, and TGF-β induce podosome formation in endothelial cells along the invasive tumor front [141,143,144,145,146]. This allows endothelial cells to reach hypoxic tumor regions, facilitating breakdown of basement membrane encapsulating the primary tumor. Once endothelial cells come into direct contact with tumor cells, endothelial cell Notch activation in response to HNSCC-derived Notch ligand Jagged1 drives capillary-like sprout formation and neovascularization of the expanding tumor mass [147]. The combined effort of endothelial cell-mediated rearrangement of the microenvironment to promote tumor cell access to the vascular network makes endothelial cells important contributors to HNSCC tumor progression.

### 3.5. Fibroblasts

The desmoplastic response is a hallmark of cancer progression, where secretion and restructuring of ECM proteins drives tumor cell proteolytic invasion and production of “tracks” for proteolytic-independent invasion modes [148,149]. Fibroblasts are specialized for this task, as they can degrade and rearrange a variety of ECM proteins including type I and IV collagens, laminin, and fibronectin [145,149,150]. Integrin α6 expression allows such cancer associated fibroblasts (CAFs) to bind the basement membrane protein laminin, enabling CAF-mediated proteolytic laminin degradation [140,151]. Expression of integrin α6 in CAFs has been linked to poor prognosis in oral cancer patients [151]. Fibroblast-mediated proteolytic cleavage of ECM proteins requires direct contact with tumor cells or binding of HNSCC secreted endothelin-1 (ET-1), leading to localization of a disintegrin and metalloprotease (ADAM)-12 and ADAM-17 at fibroblast podosomes, followed by secretion and activation of MMP-2 and MMP-9 from carcinoma cells and CAFs [34,152,153,154]. Other studies suggest that chemokine C-X-C motif receptor type 4 (CXCR4) binding to CAF-secreted chemokine C-X-C motif ligand 12 (CXCL12) initiates carcinoma derived MMP-9 secretion in the tumor microenvironment [34,155]. Regardless of the source of MMP secretion, total MMP levels and the ratio of activated MMPs to total MMP concentration compared with adjacent normal tissue positively correlates with lymph node involvement [155,156]. As a result, the HNSCC stroma is enriched in infiltrating CAFs, with the highest concentrations accumulating near the invasive front of the tumor [24,29,157]. Infiltrating CAFs have several characteristics of myofibroblasts, including enhanced proliferation and motility, expression of cytokeratins, vimentin, and α-smooth muscle actin (SMA), and secretion of MMP-2 and HGF [122,158,159]. CAF MMP secretion facilitates ECM degradation and remodeling, whereas HGF enhances HNSCC cell motility [122,158,159]. In turn, enhanced CAF proliferation and motility allows the CAF population to expand and spread in accordance with the growing invasive tumor front [122,158,159]. The adaptation of HNSCC CAFs with myofibroblast characteristics results in extracapsular tumor cell spread, increased invasion, and lymph node metastasis [160]. Orthotopic floor of mouth co-injection of HNSCC cells with CAFs or normal fibroblasts in mice indicates that CAFs contribute significantly to lymph node and distal metastatic disease [161]. The net results of fibroblasts in the tumor microenvironment is rearrangement of ECM proteins, allowing fibroblasts to lead tumor cells into surrounding tissues or paving pathways in the stroma for invasive tumor cells to follow. Additionally, TGF-β and miR-210 induced CAF senescence promotes fibroblast MMP-2 secretion and tumor cell EMT, enhancing *in vitro* tumor cell invasion [151,162,163,164]. Further evidence indicates that coinjection of tumor cells with senescent CAFs promotes xenograft engraftment and tumor growth [165,166,167]. These activities ultimately result in facilitating HNSCC metastatic progression.

## 4. Anti-Metastatic Therapeutic Approaches

While indolent primary HNSCC tumors are typically treated by surgical resection and/or radiation therapy, the treatment of invasive and metastatic disease is more complex. The development of preventative anti-metastatic therapies holds promise to broaden patient treatment options and improve survival rates. Many recent anti-metastatic treatments have been aimed at Src kinase due to the essential role Src plays in cancer cell motility and invadopodia formation, as well as the multitude of overexpressed upstream transmembrane receptors that activate Src in tumors [25,26,27]. Initial *in vitro* studies using saracatinib (AZD0530) resulted in decreased MMP-9 activation and ECM degradation in established HNSCC cell lines, and also reduced invasion in HNSCC cells lines from primary tumors and matched lymph node metastases in combination with the phospholipase C inhibitor U73122 [168,169]. Another combination study showed that saracatinib with the EGFR small molecule inhibitor gefitinib suppressed HNSCC cell invasion *in vitro* to a greater extent than either drug alone [170]. However, a subsequent Phase II trial of saracatinib resulted in no therapeutic benefit in either recurrent or metastatic HNSCC [171]. Treatment of HNSCC cell lines with the Src/Abl small molecule inhibitor dasatinib (BMS-354825) decreased migration and invasion while blocking the G1-S transition [172]. A Phase II clinical trial of dasatinib alone also failed to show clinical benefit to patients with late stage HNSCC [173]. These trial results clearly demonstrate that targeting Src is insufficient to prevent HNSCC progression, prompting the need to evaluate additional pro-invasive oncogenic targets. The activity of another oncogenic tyrosine kinase, Abl, downstream of EGFR and Src kinase facilitates invadopodia formation and promotes tumor cell invasion and metastasis [30,174,175,176,177,178]. *In vitro* treatment with the Abl family inhibitor imatinib mesylate (STI571; Gleevac) resulted in enhanced HNSCC cell invasion, opposite of what has been observed in invasive breast cancer [179]. Imatinib mesylate stimulates HNSCC shedding of heparin-binding EGF, which activates EGFR on the HNSCC cell surface, driving invadopodia formation and ECM degradation [179]. A phase II trial of imatinib mesylate and docetaxel for patients with metastatic non-small-cell lung carcinoma and HNSCC found no clinical benefit and closed early due to significant toxicity from this drug regimen [180]. Cetuximab (IMC-C225), an anti-EGFR humanized monoclonal antibody, shows multifaceted benefit in HNSCC by blocking proliferation, angiogenesis and metastasis while increasing tumor cell apoptosis [181,182,183]. Phase II clinical trials for patients with late stage HNSCC showed partial response to cetuximab alone in a small patient subset, while complete response was observed in the majority of patients when cetuximab was used in combination with cisplatin, fluorouracil, and radiotherapy [184,185]. The Erbitux in First-Line Treatment of Recurrent or Metastatic Head and Neck Cancer (EXTREME) Phase III trial showed significant increases in overall survival, progression-free survival, and response rate for the combination of cetuximab and platinum/5-fluorouralcil compared with platinum/5-fluorouracil alone [186,187,188,189]. While these trials did not directly investigate an anti-metastatic role for cetuximab, it is a promising advance in HNSCC treatment. Another study found that the potassium ionophore antibiotic salinomycin significantly inhibited growth of the cisplatin-resistant mesenchymal-like HNSCC subpopulation, likely through induction of apoptosis [101,190]. These data demonstrate a potential mechanism for targeting a drug resistant, highly mobile subpopulation that has been implicated in metastatic dissemination as well as disease recurrence [100,101,102]. While these initial studies have demonstrated some efficacy in patients with advanced disease, direct anti-invasive and anti-metastatic therapeutic targeting continues to remain elusive in HNSCC.

## 5. Conclusions

**Figure 1:**
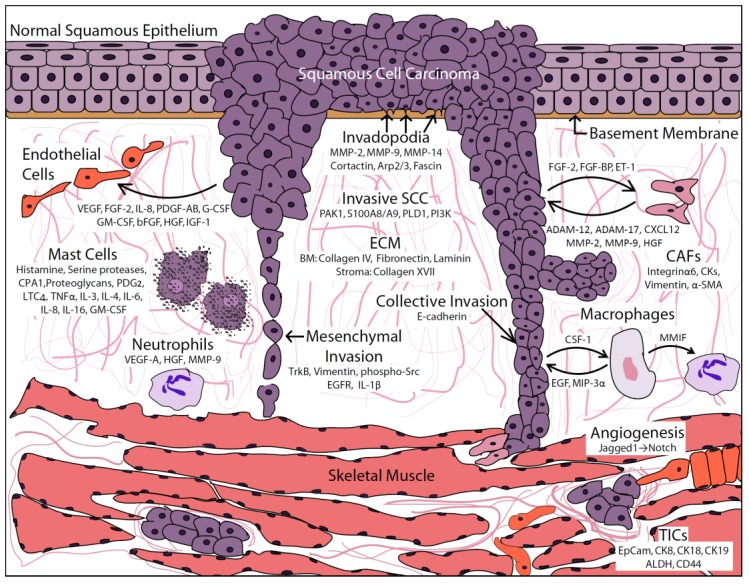
Diagram of tumor and stromal-based contributions that promote head and neck squamous cell carcinoma (HNSCC) invasion. Depicted is an invasive HNSCC tumor cell mass invading into the surrounding stroma and muscle tissue of the oral tongue. Specific cell types and their respective secreted protein contributions are detailed, demonstrating subsequent cellular responses and paracrine signaling networks. See text for additional detail.

HNSCC tumors contain a host of aberrant signaling pathways, from cytoskeletal modulation responsible for driving increased invasion to promoting tumor cell survival in the circulation. Interactions with the surrounding ECM as well as between individual tumor cells influences the ability of HNSCC cells to invade into the surrounding tissue and eventually to other parts of the body, predominantly the cervical lymph nodes. Changes in cell-cell adhesions along with alterations in cellular morphology allow HNSCC cells to undergo a variety of invasive patterns. Additionally, HNSCC cells utilize various autocrine and paracrine secreted factors in order to optimize tumor dissemination, whether through neovascularization by endothelial cells or rearrangement of ECM protein by local fibroblasts. The tumor microenvironment, depicted in Figure 1, is therefore a complex, dynamic system, complicating our understanding of tumor behavior and potential therapeutic interventions. 

Use of conventional wet-bench‒based cell and xenograft assays cannot incorporate the breath of tumor-stromal interactions that occur in patient tumors. The inability of these models to accurately guide pharmaceutical intervention development, as witnessed by the lack of successful clinical trials to date, is a testament to the complexity and difficulty of targeting HNSCC invasion and metastasis. This is likely due to the multitude of pro-invasive signaling networks in HNSCC cells and various tumor-stromal interactions. Therefore, in order to make meaningful advances in the treatment of HNSCC invasion, new model systems need to be developed that include, or at least consider, all of the intracellular, cell-cell, and cell-matrix contributions from carcinoma cells and corresponding tumor-associated stromal cells found in patient tumors.

## Abbreviations

ALDH: aldehyde dehydrogenase
ADAM-12: a disintegrin and metalloprotease 12
ADAM-17: a disintegrin and metalloprotease 17
bFGF: basic fibroblast growth factor
CAF: cancer associated fibroblast
CK: cytokeratin
CK8: cytokeratin 8
CK18: cytokeratin 18
CK19: cytokeratin 19
CSC: cancer stem cell
CTC: circulating tumor cell
CXCL12: chemokine C-X-C motif ligand 12
CXCR4: chemokine C-X-C motif receptor type 4
E-cadherin: epithelial-cadherin
ECM: extracellular matrix
EGF: epidermal growth factor
EGFR: epidermal growth factor receptor
EMT: epithelial to mesenchymal transition
EpCAM: epidermal cell adhesion molecule
ET-1: endothelin-1
F-actin: filamentous-actin
FAK: focal adhesion kinase
FGF-2: fibroblast growth factor 2
FGF-3: fibroblast growth factor 3
FGF-4: fibroblast growth factor 4
FGF-19: fibroblast growth factor 19
FGF-BP: fibroblast growth factor binding protein
G-CSF: granulocyte colony stimulating factor
GM-CSF: granulocyte macrophage colony stimulating factor
HER3: human epidermal growth factor receptor 3
HGF: hepatocyte growth factor
HNSCC: head and neck squamous cell carcinoma
IGF-1: insulin-like growth factor 1
IGF-1R: insulin-like growth factor receptor 1
IL-1β: interleukin 1β
IL-3: interleukin 3
IL-4: interleukin 4
IL-5: interleukin 5
IL-6: interleukin 6
IL-8: interleukin 8
IL-10: interleukin 10
IL-13: interleukin 13
IL-16: interleukin 16
LIMK: LIM kinase
LTC_4_: leukotriene C_4_
MAPK: mitogen activated protein kinase
MIP-3α: macrophage inhibitor protein 3α
MLC: myosin light chain
MLCK: myosin light chain kinase
MMIF: macrophage migration inhibitory factor
MMP: matrix metalloproteinase
MMP-1: matrix metalloproteinase 1
MMP-2: matrix metalloproteinase 2
MMP-3: matrix metalloproteinase 3
MMP-9: matrix metalloproteinase 9
MMP-14: matrix metalloproteinase 14
MT1-MMP: membrane type 1-matrix metalloproteinase
PAK1: p21 protein (Cdc42/Rac)-activated kinase 1
PDGF: platelet-derived growth factor
PDGF-AB: platelet-derived growth factor AB
PDGFR: platelet-derived growth factor receptor
PGD_2_: prostaglandin D_2_
PI3K: phosphoinsositide-3-kinase
TAM: tumor associated macrophage
TGF-β: transforming growth factor β
TIC: tumor initiating cell
TNF-α: tumor necrosis factor α
TrkB: tyrosine receptor kinase B
VEGF: vascular endothelial growth factor
VEGF-A: vascular endothelial growth factor A
VEGFR: vascular endothelial growth factor receptor
